# Predicting Primary and Secondary Abstinence Among Adolescent Boys and Girls in the Western Cape, South Africa

**DOI:** 10.1007/s10461-016-1438-2

**Published:** 2016-06-11

**Authors:** Sander M. Eggers, Catherine Mathews, Leif E. Aarø, Tracy McClinton-Appollis, Arjan E. R. Bos, Hein de Vries

**Affiliations:** 1 School for Public Health and Primary Care (CAPHRI), Department of Health Promotion, Maastricht University, P.O. Box 616, 6200 MD, Maastricht, The Netherlands; 2 Health System Research Unit, South African Medical Research Council, Cape Town, South Africa; 3 School of Public Health and Family Medicine, University of Cape Town, Cape Town, South Africa; 4 Adolescent Health Research Unit, Department of Psychology and Mental Health, University of Cape Town, Cape Town, South Africa; 5 Division of Mental Health, Norwegian Institute of Public Health, Oslo, Norway; 6 Department of Health Promotion and Development, University of Bergen, Bergen, Norway; 7 Faculty of Psychology and Educational Sciences, Open University, Heerlen, The Netherlands

**Keywords:** Sexual abstinence, HIV, Prevention, Adolescents, I-Change Model, South Africa

## Abstract

Two of the most effective health behaviours with regard to HIV prevention are condom use and sexual abstinence. While determinants of condom use among sub-Saharan African adolescents have been studied extensively, factors related to abstinence have received far less attention. This study identified socio-cognitive determinants of primary and secondary abstinence intentions and of early sexual activity. This study also assessed whether these factors had a direct or indirect association with intentions to abstain from sex. A longitudinal design was used in which 1670 students (age 12–16) of non-private South African high schools filled in a questionnaire, with a follow-up after 6 months, concerning sexual abstinence, attitudes, social norms, self-efficacy, risk perception and knowledge. Logistic and linear regression analysis with latent factors was used to assess determinants of intentions and abstinence, and structural equation modelling was used to assess indirect effects. Results showed that among sexually inactive students, social norms predicted the intention to abstain from sex in the next 6 months. Among sexually active students, reporting less disadvantages of abstinence predicted the intention to abstain. Sexual activity at follow-up was predicted by attitudes and intention among sexually inactive girls, and by knowledge among sexually inactive boys. No predictors were found for sexually active adolescents. Structural equation modelling further showed that risk perception was indirectly related to intentions to abstain from sexual intercourse. We conclude that addressing socio-cognitive factors in order to motivate adolescents to delay sex is more likely to be successful before they experience sexual debut. In addition, this study shows that the effect of increasing risk perceptions, a strategy often applied by parents and HIV prevention programmes, is to a large extent mediated by more proximal cognitive factors such as attitude. Research is needed to identify factors that influence the execution of intentions to abstain from sex.

## Background

In South Africa, approximately 5.7 million people are living with HIV and incidence rates range from 0.5 % in the Western Cape till 2.3 % in Kwazulu-Natal [1]. Although recent estimates show that the spread of the epidemic is slowing down, there is still a strong need for effective interventions as outlined by the South Africa National Strategic Plan for HIV, STI’s and TB [2]. One of the four key strategic objectives mentioned is the prevention of new HIV and STI infections by at least 50 %. To reach this objective, the National Strategic Plan emphasises the importance of targeting behavioural and socio-cognitive factors such as early sexual debut, condom use, knowledge and risk perception, in addition to more structural barriers such as poverty and access to sexual and reproductive health services.

An extensive worldwide review of the effectiveness of sexual and HIV education programmes by Kirby et al. [3] concludes that more research is needed into the mediating role of socio-cognitive factors, such as risk perception, to broaden our understanding of motivational processes. New research should investigate the interdependency between factors and should assess how factors like attitudes and self-efficacy could mediate effects of interventions or even the effects of other socio-cognitive factors on behaviour. Recent studies have corroborated this statement and have shown that the assessment of mediating and moderating pathways to understand behaviour with techniques like structural equation modelling is becoming increasingly popular [4–7]. As such, detailed knowledge of motivational pathways could lead to more effective interventions and the development of more informative socio-cognitive theories.

In the past decade, several comprehensive theories that integrate evidence-based elements from theories such as the Theory of Planned Behaviour [8], The Health Belief Model [9] and Socio-Cognitive Theory [10] have been proposed. The use and combination of such theories to guide development and pinpoint key determinants is recommended [11–14]. One of the integrative models that have been applied successfully in South African sexual risk behaviour studies is the I-Change Model [15, 16]. The I-Change Model is distinct from the models described above, in that it postulates a series of phases to understand the behaviour change process, and adds a pre- and a post-motivational phase to the motivational phase postulated by the models described above [17]. The development of an awareness of the problem and one’s own health behaviour is central in the premotivational phase, and determined by factors such as knowledge, risk perceptions and cognizance (i.e. an accurate perception) of one’s own behaviour. If a person is aware of the problem, the associated health risks and his or her (lack of) health behaviour (e.g. not using condoms), a person will be more motivated to process information about motivational factors such as the pros and cons of the desired behaviour (the attitude), social influence beliefs, and self-efficacy, factors also outlined by the Theory of Planned Behaviour [8]. Together, these motivational factors predict the intention to engage in the health behaviour. If the person is motivated to engage in the health behaviour, the translation of intention into behaviour is determined by self-efficacy, levels of action planning, plan enactment and the level of barriers that are encountered (e.g. no access to condoms). The results of a previous study which assessed condom use among South African adolescents showed that the I-Change Model and its motivational pathways exhibited good model fit and adequate proportions of explained variance [18]. The results also confirmed a basic postulate of the I-Change Model, in that the influences of knowledge and risk perception were mediated by motivational factors.

Relatively few studies, however, have assessed socio-cognitive correlates of primary and secondary abstinence among sub-Saharan adolescents [19, 20] and even fewer have used prospective designs [21, 22]. While primary abstinence is defined as having no previous oral or penetrative sexual experience, secondary abstinence is defined as a period of no oral or penetrative sex after sexual initiation has already occurred. The findings of previous studies indicated that self-efficacy to refuse sex, self-efficacy to negotiate sex, attitudes towards delaying sex, social norms, perceptions that most friends are having sex (social modelling), and intentions to delay sex were all significantly associated with adolescent sexual activity [19–22]. Similarly, attitudes to delay sex, social norms and self-efficacy were significantly associated with intentions to delay sex among samples of sexually active (i.e. secondary abstinence intentions) and non-sexually active adolescents (i.e. primary abstinence intentions).

Yet, less is known concerning factors that are important for the premotivational phase. Consequently, the role of awareness factors such as knowledge and risk perception is less clear. According to a meta-analysis by Sheeran et al. [23] of 121 condom use studies, knowledge and risk perception have only small correlations with condom use (weighted r < 0.10). More recent studies in sub-Saharan Africa corroborate this and were unable to find strong associations between risk perception and behaviour or intentions [21, 24–26]. These findings are unexpected, since theories such as the Health Belief Model and Protection-Motivation theory, assume that risk perception is directly associated with intentions or behaviour [9, 27]. Similarly, the Information-Motivation-Behavioral Skills model (IMB) assumes that knowledge (i.e. information) has both direct and indirect associations with behaviour [28]. A potential explanation for this discrepancy between theory and empirical data is that knowledge and risk perception may not be directly, but rather indirectly related to intentions and behaviour, as hypothesised by the I-Change Model and IMB model [17, 28].

Since little is known about the socio-cognitive predictors of primary and secondary abstinence in South Africa, the main goal of this study is to assess socio-cognitive correlates and adjusted predictors of early sexual activity and of intentions to abstain from sex (both primary and secondary abstinence). The results will be presented for males and females separately since previous research has shown considerable gender differences in the predictive value of socio-cognitive factors with regard to sexual behaviour [29, 30, 41]. Second, we will use structural equation modeling to assess whether the socio-cognitive factors knowledge and risk perception are directly or indirectly longitudinally associated with intentions to abstain from sex.

## Method

### Sample and Procedures

Data was gathered using self-report questionnaires as part of the PREPARE study [16]. The PREPARE study aimed to promote sexual and reproductive health by focusing on (1) delaying sexual debut or delaying sexual intercourse for those in new relationships, and (2) by promoting condom use among the sexually active. For this study, only data from the control group of the PREPARE trial was used. The experimental group consisted of different schools which were outside the immediate vicinity of the control schools to avoid any accidental exposure to the intervention. A full description of the PREPARE programme is given by Aarø et al. [16]. Informed consent was obtained from all participants and school authorities. Paper and pen questionnaires were filled out by the adolescents in class during regular school sessions at the first survey (March 2013; T1) and at follow-up after 6 months (T2). All adolescents (*N* = 1670) were in grade 8 of 20 randomly chosen public schools in the Western Cape, South Africa. Only PREPARE project staff was present during data collection and adolescents could hand in their completed questionnaire in a sealed envelope to ensure confidentiality.

The PREPARE study was approved by the Western Norway Regional Committee for Medical and Health Research Ethics. Separate ethics approvals were provided by relevant committees in each of the African sites: Western Cape, South Africa: Human Research Ethics Committee, Faculty of Health Sciences, University of Cape Town; Dar es Salaam, Tanzania: The Senate Research and Publications Committee of the Muhimbili University of Health and Allied Sciences.

### Measurements

The questionnaire was based on previous sexual risk behaviour research and focus interviews with the target group and key-informants (e.g. parents, teachers, health care workers). The questionnaire included three languages to aid comprehension (i.e. English, Zuid-Afrikaans and Xhosa). Besides socio-cognitive factors and socio-economic status, age and gender of each participant was recorded.

#### *Socio*-*economic status (SES)*

Participants were asked to indicate which of eight household assets they had access to at home. Household assets queried were tap water, toilet, electricity, landline (telephone), a mobile telephone, fridge, TV and a car. Participants could respond with either ‘yes’ or ‘no’. Affirmative answers were summed to create a family affluence scale [31].

#### Pros of Delaying Sex

The perceived pros (advantages) of delaying sex were assessed by four items and responses were captured using a 5-point Likert scale consisting of ‘strongly disagree (1); disagree (2); not sure (3); agree (4); strongly agree (5)’. The four items were ‘waiting until I am older before I have sex will: help me achieve my life’s goals; will help prevent me from getting hurt emotionally; will please my parents, will lower my risk of getting HIV’. Scores were averaged to form a scale and all factor loadings were higher than 0.50 (minimum acceptable value is 0.40 according to Stevens [32]; Cronbach’s α = 0.72).

#### Cons of Delaying Sex

The perceived cons (disadvantages) of delaying sex were assessed by four items and responses were captured using the same 5-point Likert scale as for the pros. The four items were ‘waiting until I am older before I have sex will: make me look old-fashioned; will be frustrating for me; will make my partner frustrated with me; will make me look unsuccessful’. Scores were averaged to form a scale and all factor loadings were higher than 0.50 (Cronbach’s α = 0.81).

#### Social Norm

Perceived norms of delaying sex were assessed by four items and responses were captured using the same 5-point Likert scale as for the pros. The four items were ‘most of my friends think that I should wait until I am older before I have sex; most of parents/caregivers think I should wait until I am older before I have sex; most of my other family members think that I should wait until I am older before I have sex; my boyfriend or girlfriend thinks that I should wait until I am older before I have sex’. Scores were averaged to form a scale and all factor loadings were higher than 0.50 (Cronbach’s α = 0.76).

#### *Self*-*efficacy*

Self-efficacy to delay sex was assessed by four items and responses were captured using a 5-point Likert scale consisting of ‘very difficult for me (1); difficult for me (2); not sure (3); easy for me (4); very easy for me (5)’. The four items were ‘if I have been drinking alcohol, waiting until I am older before I have sex is…; if my partner is older than me, waiting until I am older before I have sex is…; if someone offers me money or gifts, waiting until I am older before I have sex is…; if I am deeply in love, waiting until I am older before I have sex is…’. Scores were averaged to form a scale and all factor loadings were higher than 0.50 (Cronbach’s α = 0.80).

#### Knowledge

Knowledge about HIV and safe sex was assessed by eight items and responses were captured using three options ‘Yes; no; I don’t know’. The eight items were ‘if you have sex only once, with a person who is HIV positive, can you become infected with HIV? If you kiss with a person who is HIV positive, can you become infected with HIV? If you have anal sex with a person who is HIV positive, can you become infected with HIV? Can a person who looks strong and health look be HIV positive; does a condom have an expiry date; Is it true that the only time that a person should use a condom is when they have sex with someone for the first time? When a girl uses contraceptive pills or the injection for family planning, does this protect her against sexually transmitted infections? Should a man leave a bit of air at the top of the condom when putting it on?’. Items were recoded into correct (1) and incorrect answers (0) and subsequently averaged to get a score that reflects the average proportion of correct answers (ranging from 0 = none correct, to 1 = all questions correct).

#### Risk Perception

Risk perceptions towards getting STI’s and HIV were assessed by four items pertaining to susceptibility and severity. Response options were ‘very low (1), low (2), not sure (3), high (4), very high (5)’ for susceptibility, and ‘not serious (1), a bit serious (2), not sure (3), serious (4), very serious (5)’ for severity. The items related to susceptibility were ‘If I do not use a condom when having sex, my risk of getting a sexually transmitted infection will be…; if I do not use a condom when having sex, my risk of HIV infection will be…’. Items related to severity were ‘if I got an STI, I would find this…; if I was infected with HIV I would find this…’. Scores were averaged to form a risk perception scale with all factor loadings higher than 0.50 (Cronbach’s α = 0.77).

#### Intention

The intention to abstain from sex was assessed by three items and responses were captured using the same 5-point Likert scale as for the pros. The three items were ‘I intend to have sex within the next month; I intend to have sex within the next 6 months; I intend to have sex within the next year’. Scores were averaged to form a scale and all factor loadings were higher than 0.50. All responses were inversely coded to reflect the intention to abstain from sex (Cronbach’s α = 0.88). The reason these intention items were formulated in an affirming way (e.g. ‘I intend to have sex’), was to make them more understandable for our target group and for them to be less suggestive. Pilot testing of the questionnaire specifically showed that this formulation led to a better understanding of the question, since ‘intending not to do something’ was considered to be contradictive by some adolescents.

#### Sexual Behaviour

At T1, three items assessed whether adolescents ever had sex, namely ‘have you ever had vaginal sex; have you ever had anal sex; have you ever had oral sex’. Definitions of each type of sex were given in the questionnaire. Response options were ‘yes’ or ‘no’, and an affirmative answer on any of the three questions was recoded into ‘sexually active (1)’. Three consecutive negative answers on these questions was recoded into ‘not sexually active (0)’. At follow-up after 6 months, the same questions and response options were used, but with a slightly different formulation specifying the time frame, namely ‘during the last 6 months, did you have vaginal sex; did you have anal sex; did you have oral sex’.

### Statistical Analyses

Data entry and descriptive analyses were done using SPSS version 20. Chi square tests and analyses of variance (ANOVA) were used to describe the sample. Correlations and the proposed pathways of the I-Change model were modelled using Mplus version 7.3 [33]. Indirect effects were assessed using the Delta method and all standard errors in the regression models were adjusted for the nested structure of students within schools using the cluster command available in Mplus [33]. Model fit was assessed using the Comparative Fit Index (CFI) and the Root Mean Square Error of Approximation (RMSEA). Good model fit is indicated by CFI values higher than 0.95 and RMSEA values below 0.06 [34] and adequate model fit is indicated by CFI values over 0.90 and RSMEA values below 0.08 [35]. Items that were used as indicators for latent variables were defined as categorical and the Weighted Least Squares Mean and Variance adjusted estimator (WLSMV) was used to calculate coefficients [36]. Reported regression coefficients are unstandardized, since nearly all socio-cognitive factors used the same 5-point Likert scales, and effects were considered significant when *p* < 0.05.

## Results

### Sample Characteristics

The adolescents (*N* = 1670) were 13.6 years of age on average (SD = 1.01; range 12-16), were predominantly female (61.6 %, *N* = 1029/1670), had access to an average of 6.06 household assets (SD = 1.65; range 1-8). Independent samples t-tests showed that boys were slightly, but statistically significantly older than girls (Δ age = 0.36); had lower intentions to delay sex (Δ intention = 0.48); less risk perceptions (Δ risk perception = 0.18); perceived less pros of abstinence (Δ pros = 0.16); perceived more cons of abstinence (Δ cons = 0.33); perceived less social norms with regard to abstinence (Δ social norms = 0.23); and had less self-efficacy to abstain from sex in comparison to the girls (Δ self-efficacy = 0.37) (all *p* < 0.01). Boys also reported more often that they were sexually active at T1 than girls (35.8 % and 9.8 % respectively; *Χ* ^2^ (1, 1667) = 166.9; *p* < 0.001).

The total proportion of adolescents that were sexually active at T1 was 20.3 % (*N* = 339/1670), and 36.7 % (*N* = 121/330) of the sexually active adolescents were intending to stay abstinent in the upcoming year (i.e. secondary abstinence). Of the sexually inactive adolescents, 73.8 % (*N* = 932/1263) were intending to stay abstinent in the upcoming year (i.e. primary abstinence).

After 6 months (T2), 29.0 % of the inactive boys (*N* = 117/403) had experienced sexual debut, in comparison to 14.5 % of the inactive girls (*N* = 135/928) (*Χ* ^2^ (1, 1331) = 38.4; *p* < 0.001). Logistic regression analysis showed that this difference between boys and girls was significant even after adjustment for age and other differences at T1 (OR 2.85; p < 0.001). Among adolescents that were already sexually active at T1, 37.8 % (*N* = 128/339) reported to not have had sex during the 6 months between T1 and T2 (see Table 1).

**Table 1:** Means and simple correlations of socio-cognitive factors with intentions to abstain and sexual activity

	Sexually non-active adolescents (N = 1331)								Sexually active adolescents (N = 339)			
	Boys (N = 403)				Girls (N = 928)							
	Mean (SD)	Intention T1	Intention T2	Sexual activity T2	Mean (SD)	Intention T1	Intention T2	Sexual activity T2	Mean (SD)	Intention	Intention	Sexual activity T2
										T1	T2	
Baseline T1												
Intention to abstain	4.10 (1.13)	–	0.49*	−0.04	4.42 (0.98)	–	0.50*	−0.14*	3.32 (1.27)	–	0.51*	0.03
Age	13.76 (1.03)	−0.28*	−0.34*	−0.19*	13.51 (0.89)	−0.19*	−0.22*	0.20*	14.12 (1.36)	−0.24*	−0.26*	0.28*
SES	6.23 (1.49)	0.04	0.30*	−0.14*	6.06 (1.63)	0.11*	0.08*	−0.09	5.67 (1.73)	0.05	0.09	0.02
Pros	3.90 (0.86)	0.07	0.06	0.03	4.05 (0.83)	0.13*	0.14*	−0.12*	3.95 (0.81)	0.06	0.16*	−0.20*
Cons	2.73 (1.03)	−0.29*	−0.22*	0.11	2.40 (0.99)	−0.24*	−0.22*	0.11	2.85 (1.02)	−0.36*	−0.21*	−0.12
Social norms	4.29 (0.73)	0.18*	0.16*	0.03	4.46 (0.68)	0.29*	0.28*	0.01	4.07 (0.83)	0.12*	0.22*	−0.21*
Self-efficacy	2.96 (0.98)	0.04	0.1	−0.14	3.28 (1.09)	0.25*	0.17*	−0.03	2.68 (0.98)	0.19*	0.13*	−0.01
Risk perception	3.78 (1.02)	0.28*	0.24*	0.06	3.93 (0.89)	0.36*	0.28*	0.04	3.51 (1.12)	0.25*	0.12	0.19
Knowledge^a^	41.70%	0.03	0.02	0.14*	41.60%	0.09*	0.14*	0.13*	45.50%	0.01	0.01	0.11
Sexual activity	0%	–	–	–	0%	–	–	–	100%	–	–	–
Follow-up T2												
Intention to abstain	3.96 (1.21)	0.49*	–	−0.53*	4.37 (0.97)	0.50*	–	−0.43*	3.55 (1.20)	0.51*	–	−0.34*
Pros	3.92 (0.80)	0.08	0.01	0.02	4.14 (0.78)	0.23*	0.19*	0.11	4.01 (0.82)	0.18*	0.14*	−0.08
Cons	2.54 (1.01)	−0.25*	−0.32*	0.08	2.23 (0.93)	−0.27*	−0.34*	0.01	2.65 (1.02)	−0.27*	−0.29*	−0.09
Social norms	4.32 (0.75)	0.20*	0.19*	−0.1	4.48 (0.63)	0.28*	0.43*	−0.25*	4.05 (0.83)	0.21*	0.21*	−0.28*
Self-efficacy	3.02 (0.97)	0.14	0.14*	−0.08	3.44 (1.05)	0.26*	0.29*	−0.14*	3.04 (0.97)	0.25*	0.26*	−0.30*
Risk perception	3.82 (0.97)	0.20*	0.22*	0.02	4.00 (0.86)	0.26*	0.24*	0.09	3.82 (0.97)	0.25*	0.14	0.16
Knowledge^a^	41.20%	0.06	0.1	0.18*	42.10%	0.08*	0.07*	0.25*	47.00%	0.07	0.01	0.30*
Sexual activity	29.00%	−0.04	−0.53*	–	14.50%	−0.14*	−0.43*	–	62.20%	−0.03	0.34*	–

^a^Defined as the proportion of correctly answered questions

* *p* < 0.05

### Longitudinal Correlates of Sexual Activity and Intentions to Abstain

Means and correlations are presented in Table 1. Factors that were associated with the intention to abstain from sex at T1 were low levels of perceived cons of abstinence; strong perceived social norms towards abstinence; and strong risk perceptions towards sexually transmitted infections. This was true for both sexually active and inactive adolescents. Self-efficacy to delay sex was significantly associated with the intention to abstain among girls and among sexually active adolescents, but not among sexually inactive boys. Knowledge of HIV and condom use was only significantly related to intentions to abstain among girls. Similar results were obtained with regard to intentions to abstain at follow-up (see Table 1).

Factors that were associated with actual sexual activity at follow-up (T2) differed considerably between groups. For sexually inactive boys, higher knowledge concerning HIV and condom use was significantly associated with sexual activity at follow-up. For sexually inactive girls, lower intentions to abstain; perceiving less pros of abstinence; and higher levels of knowledge was significantly associated with sexual activity at follow-up. It must be noted that the associations were relatively small in terms of effect size (all *r*’s between 0.10 and 0.15) [37]. Among sexually active adolescents, factors that were significantly associated with sexual activity after 6 months (T2) were less perceived pros of abstinence and weaker social norms with regard to sexual abstinence (see Table 1).

### Adjusted Predictors of Primary and Secondary Abstinence Intentions

To assess which socio-cognitive factors may explain intentions to stay sexually abstinent among adolescents, intentions to stay abstinent at T2 were regressed on the pros, cons, social norms, self-efficacy, knowledge, and risk perception at T1. Results are presented in Table 2 and showed that social norms concerning abstinence and risk perceptions concerning STI’s were significant predictors of intentions to abstain from sex among inactive adolescents. It must be noted however, that among boys, the effect of risk perception was only marginally significant (*p* = 0.059). Another factor that was strongly, but negatively related to the intention to abstain from sex was whether these adolescents experienced sexual debut during the 6 months following T1.

**Table 2:** Adjusted socio-cognitive predictors of intentions to not have sex at T2

	Sexually inactive adolescents				Sexually active adolescents	
	Boys		Girls		B	95 % CI
	B	95 % CI	B	95 % CI		
Baseline T1						
Pros	−0.06	−0.22 to 0.09	−0.14	−0.37 to 0.09	0.07	−0.10 to 0.24
Cons	−0.1	−0.24 to 0.03	−0.08	−0.24 to 0.08	−0.22**	−0.37 to 0.05
Social norms	0.19*	0.01 to 0.38	0.29**	0.10 to 0.49	0.11	−0.03to 0.25
Self-efficacy	−0.06	−0.30 to 0.17	0.02	−0.07 to 0.11	0.01	−0.14 to 0.16
Risk perception	0.22	−0.02 to 0.47	0.22**	0.06 to 0.37	−0.05	−0.29 to 0.20
Knowledge^a^	0.19	−0.47 to 0.86	0.29	−0.10 to 0.69	0.15	−0.28 to 0.59
Sexually active at T2	−0.68***	−0.88 to 0.49	−0.73***	−0.92 to 0.53	−0.60***	−0.75 to 0.45
R^2^	0.3		0.21		0.24	

* *p* < 0.05; ** *p* < 0.01; *** *p* < 0.001

^a^Defined as the proportion of correctly answered questions

Among those who were already sexually active at T1, perceiving less cons of abstinence was significantly related to sexual activity at follow-up. In addition, whether they had sex in the previous 6 months predicted their future intention to abstain. Proportions of explained variance were moderate and ranged from 21 to 30 % (see Table 2). These analyses were subsequently repeated with age and SES as covariates in order to adjust for their potential influence. Results were similar, with no differences in terms of significance and only minor changes in effect sizes. In addition, being younger predicted the intention to abstain among inactive boys (B = −0.15, SE = 0.05, *p* = 0.003); among inactive girls (B = −0.11, SE = 0.04, *p* = 0.013); and among adolescents already sexually active at T1 (B = −0.10, SE = 0.03, *p* = 0.003). Higher levels of SES predicted intentions to abstain only among inactive boys (B = 0.10, SE = 0.03, *p* < 0.001).

### Adjusted Predictors of Sexual Activity

To assess which socio-cognitive factors may explain sexually abstinence among adolescents, sexual activity between T1 and T2 (measured retrospectively at follow-up) was regressed on the pros, cons, social norms, self-efficacy, knowledge, and risk perception at T1. Results are presented in Table 3 and showed that among sexually inactive boys, only knowledge predicted whether they had sex in the following months, with the full set of socio-cognitive variables explaining 8.5 % of the variance in behaviour. Among inactive girls, lower intentions to abstain, less perceived pros and more perceived cons of abstinence were significant predictors of sexual activity between T1 and T2, with the model explaining 7.7 % of the variance. Among adolescents that were already sexually active at T1, no socio-cognitive variable was found to be significantly associated with sexual activity between T1 and T2, with the model explaining 12.6 % of the variance (see Table 3).

**Table 3:** Adjusted socio-cognitive predictors of sexual activity

	Sexually non-active adolescents				Sexually active adolescents	
	Boys		Girls		OR	95 % CI
	OR	95 % CI	OR	95 % CI		
Baseline T1						
Intention to abstain	1.05	0.84–1.31	0.85*	0.74–0.99	1.09	0.87–1.38
Pros	0.92	0.67–1.27	0.79*	0.64–0.96	0.78	0.58–1.05
Cons	1.24	0.89–1.73	1.25*	1.01–1.56	0.89	0.62–1.26
Social norms	1.11	0.81–1.54	0.84	0.66–1.08	0.84	0.65–1.09
Self-efficacy	0.79	0.55–1.12	0.92	0.80–1.06	0.81	0.55–1.20
Risk perception	1.27	0.99–1.62	0.98	0.78–1.24	1.48	0.89–2.49
Knowledge^a^	2.05*	1.06–3.95	1.48	0.82–2.67	1.35	0.52–3.52
R^2^	0.09		0.08		0.13	

* *p* < 0.05; ** *p* < 0.01; *** *p* < 0.001

^a^Defined as the proportion of correctly answered questions

The same analyses were repeated with age and SES as covariates in order to adjust for their potential influence. Results showed no differences in significance of the socio-cognitive factors and only minor changes in effect size. In addition, being older significantly predicted sexual activity at follow-up for girls (B = 0.21, SE = 0.06, *p* = 0.001) and for adolescents already sexually active at T1 (B = 0.23, SE = 0.07, *p* = 0.002). SES was not significantly related to sexual activity at follow-up.

### Cross-Lagged Effects and Indirect Effects of Knowledge and Risk Perceptions

The I-Change model suggests that the effects of knowledge and risk perceptions on intentions will be mediated to a large extent by motivational factors (e.g. attitude, social influences, self-efficacy and intention). To test this, cross-lagged models were formed with each socio-cognitive factor at T2 regressed on its T1 counterpart and on each other socio-cognitive factor.

**Fig. 1:**
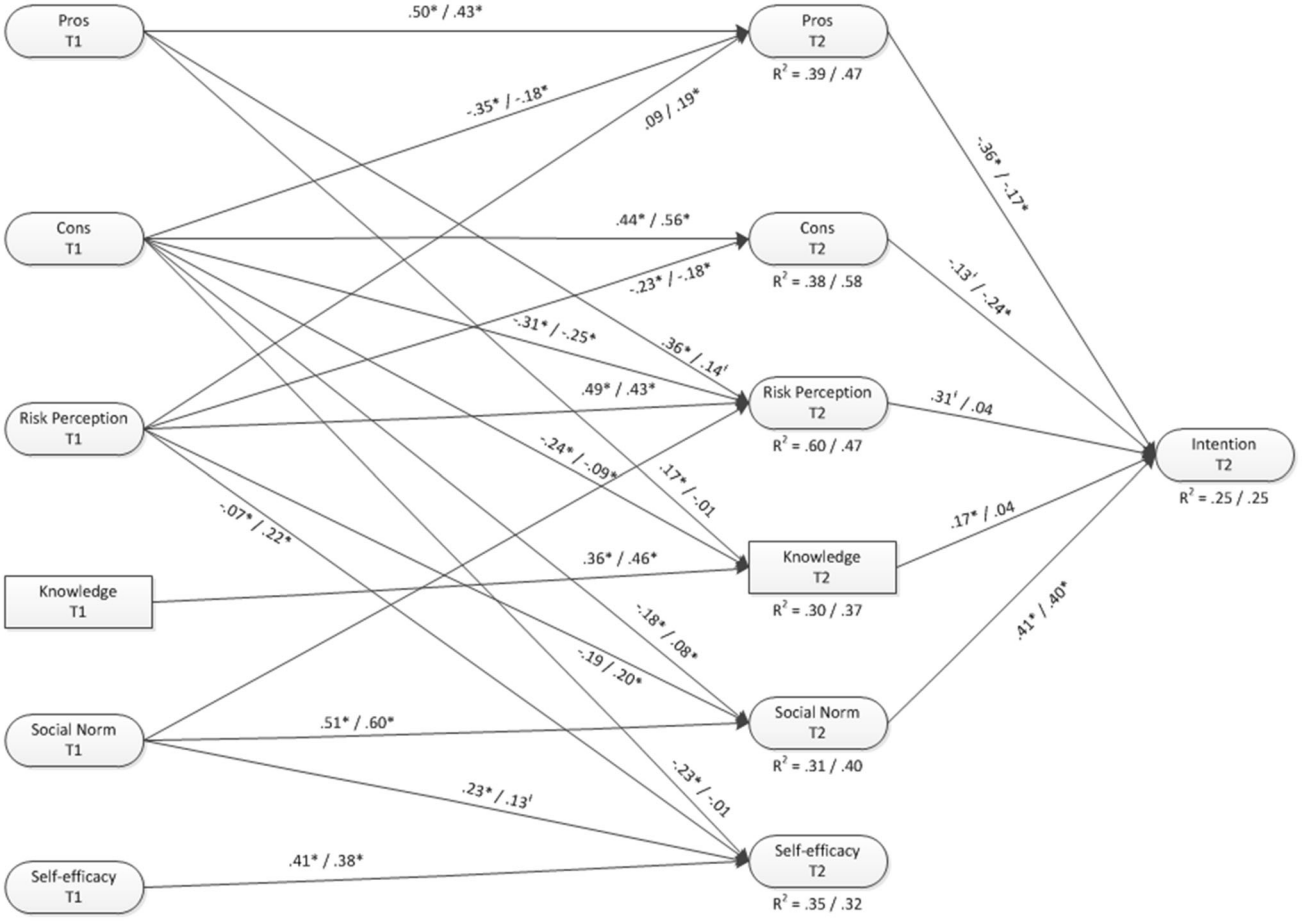
Cross-lagged socio-cognitive model predicting primary abstinence intentions (non-sign pathways are not shown). Results are shown separately for males (first) and females (secondly)

First, among the sample of sexually inactive adolescents, the results showed that both risk perception and cons of abstinence had considerable cross-lagged effects, implying that they contribute significantly to the formation of motivational beliefs (see Fig. 1). More specifically, the results indicated that reporting less cons of abstaining from sex or reporting higher risk perceptions towards STI’s at T1 was significantly related to more favourable attitudes towards abstinence, higher risk perceptions, more knowledge, higher self-efficacy and stronger social norms towards abstinence at follow-up after 6 months. Interestingly, the relationship between cons and norms among girls was positive, indicating that more perceived disadvantages at T1 was associated with more favourable social norms towards abstinence at follow-up. Most likely, this was caused by covariance with other factors (i.e. suppressor effect), since the simple correlation between the two factors was negative (*r* = −0.12) [38]. Another notable difference between boys and girls was the prediction of self-efficacy. While cons significantly predicted self-efficacy among boys this was not the case for girls. Yet, T1 risk perception significantly predicted later self-efficacy in girls but not in boys.

**Fig. 2:**
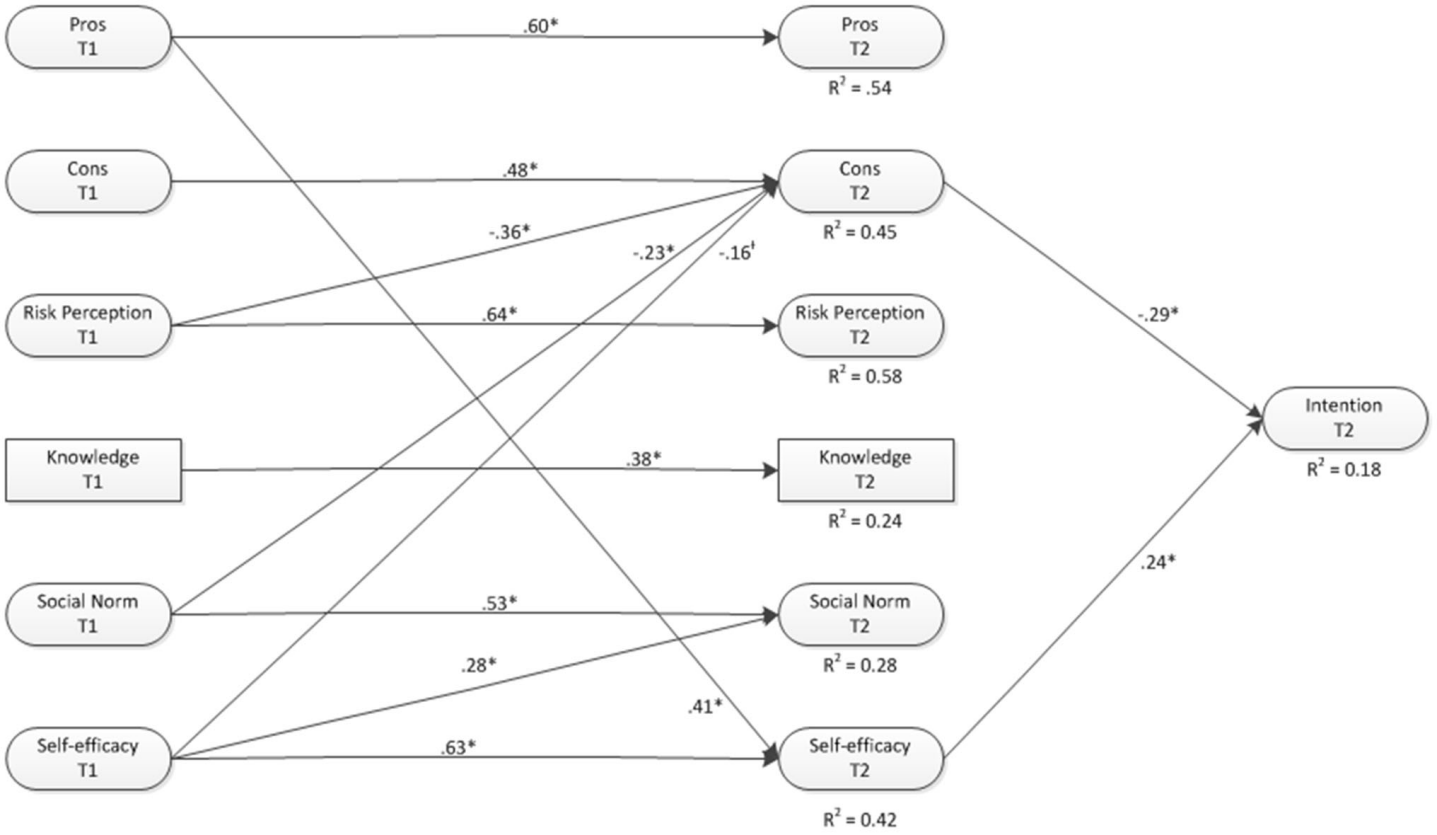
Cross-lagged socio-cognitive model predicting secondary abstinence intentions (non-sign pathways are not shown)

Second, among the sample of sexually active adolescents model fit was somewhat lower than for the sexually inactive adolescents and only a few cross-lagged pathways were significant (see Fig. 2). Higher risk perceptions and stronger social norms predicted less perceived cons at follow-up. Higher levels of self-efficacy at T1 predicted stronger social norms at follow-up and perceived pros of abstinence predicted self-efficacy at follow-up.

Indirect pathways from risk perception and knowledge via pros, cons, social norm and self-efficacy to intention to abstain were assessed for both models (i.e. sexually active and sexually non-active adolescents). Results from the sample of sexually inactive boys indicated no significant mediation effects of risk perception or knowledge. For sexually inactive girls, the results indicated that effects of risk perception on intention were significantly mediated via perceived pros (effect size = 0.03; *p* = 0.04), perceived cons (effect size = 0.04; *p* = 0.03) and social norms (effect size = 0.08; *p* = 0.03). Results from the sample of adolescents that were already sexually active at T1 indicated that effects of risk perception on intention were significantly mediated via the perceived cons only (effect size = 0.11; *p* = 0.02). No significant mediation effects were found for knowledge.

## Discussion

The first goal of this longitudinal study aimed at identifying the socio-cognitive predictors and correlates of primary abstinence, secondary abstinence and of early sexual activity among adolescents in the Western Cape, South Africa. With regard to primary abstinence intentions, our findings indicated that attitude, or more specifically the perceived negative consequences of abstinence (i.e. cons), and social norms, were moderately and consistently correlated with intentions to abstain from sex but not with sexual activity itself. For girls, additional correlates of the intention to abstain were pros, self-efficacy, risk perception and knowledge. After adjustment for covariance, only the cons, social norms and risk perception remained significant predictors. In addition, having had sex at T2 was strongly associated with intentions to have sex at T2. Moreover, the size of the association was similar for students with or without sexual experience at T1, implying that sexual debut may lead to general intentions to have sex in the near future. Overall, proportions of explained variance were relatively low and ranged from 21 % for girls, to 30 % for boys. Other studies in sub-Saharan Africa however, have shown similar estimates [21, 22].

With regard to sexual activity, correlates differed considerably between the three groups. For inactive boys, a higher level of knowledge at T1 was associated with experiencing debut in the following months. This may be indicative of the fact that those who are prone to debut are already showing interest in sexual topics. Since causality cannot be implied from the methodology used in this study, it seems likely to assume that this is the case, instead of assuming that higher knowledge actually leads to increased sexual behaviour. For girls, attitudes and intentions at T1 were moderately correlated with sexual debut, but not with social norms. It must be said however, that attitudes, social norms and intentions were all highly intercorrelated. Given the inexperience of these adolescents with sex, they may have adopted existing social norms surrounding sexual debut and internalized them as personal attitudes. Strong parenting, peer pressure and education may also play a key role in this process. In addition, the fact that risk perception was not longitudinally associated with intention or behaviour among sexually active adolescents implies that the preventive strategy often implemented by parents and teachers, namely stimulating perceptions of fear, is probably more effective among non-sexually active adolescents than among sexually active adolescents. This is especially relevant for sub-Saharan Africa where stimulating fear to avoid early sexual activity is common practice [39]. After sexual debut, adolescents could potentially benefit more from skills-based strategies, such as assertiveness training and communication skills, in order to bridge the gap from intention to behaviour.

For those adolescents that were already sexually active, pros of abstinence and social norms that support abstinence were both associated with less sexual activity in subsequent months. After adjustment for covariance, no significant predictor was found, with the model explaining 12 % of the variance in behaviour. Since statistical power was considerably smaller in the relatively small sample of sexually active adolescents, it is difficult to draw conclusions. The lack of explained variance in all models that assessed sexual behaviour at follow-up does however suggest that socio-cognitive models may lack the appropriate ingredients to explain sexual behaviour among young adolescents. Alternatively, it may be that because of their age, cognitions surrounding sex may not have been fully developed or may be difficult to express. Future studies that assess potential moderation effects on the association between intention and sexual behaviour are recommended.

Finally, with regard to indirect effects and cross-lagged associations, it was shown that the motivational pathways as proposed by the I-Change model fit the data rather well. Our findings are also in line with previous studies, showing weak associations between sexual risk behaviour and knowledge [18, 21, 25, 26], and weak associations between sexual risk behaviour and risk perceptions [23, 40–42]. The current study extends these earlier findings by showing that the influence of risk perception is likely to be mediated by motivational factors, thereby indicating that the absence of significant correlations does not mean that such factors are unimportant for behaviour change. The findings show that addressing risk perceptions may be associated with changes in attitudes, which over time may influence intentions and ultimately behaviour. Knowledge however, was not mediated via any motivational factor, and did not show any significant cross-lagged effects. This implies that knowledge is a rather distinct concept that determines behaviour independently of other socio-cognitive factors. In contrast, attitude, risk perception, and social norm were more closely related, as was apparent from the large amount of significant cross-lagged pathways for these factors. This is quite relevant for future interventions since it shows that increasing risk perceptions could lead to changes in attitudes and perceived social norms, ultimately leading to a change in motivation and potentially behaviour. Eliciting changes in knowledge about HIV and condom use will however, not have such an effect on attitudes and perceived social norms. This is line with previous research which showed that differences in knowledge do not necessarily explain motivation or behaviour [18, 21, 25, 43].

This study is subject to certain limitations. First and foremost, sexual behaviour is difficult to measure, and the self-report responses used in the current study could have been influenced by social desirability. Likewise, as adolescents became more familiar with the topic during the period of the study, their conceptions and interpretations of certain items concerning sex may have changed. In addition, for older adolescents, frequency measures of sexual activity would be advisable since they provide more statistical detail and because sexual frequency is strongly associated with the risk of HIV-infection. In the current study, scores on the frequency measures were very much skewed towards zero with the tail consisting of cases with excessive amounts of sex, possibly explained by the boasting about sex among certain young adolescents. The authors were very much unsure of these findings and chose to use the dichotomized version of the question instead (e.g. ‘have you ever had sex?’). Second, the sample size used was substantial, but the number of sexually active adolescents at T1 was relatively small, prohibiting the application of differential analyses for sexually active boys and girls. Some of the results on this aggregated sample may therefore lack specificity, and should be regarded with caution.

In conclusion, future interventions that promote sexual abstinence could benefit from the findings of this study by addressing social norms, attitudes and risk perceptions in order to influence motivations to stay abstinent during adolescence. Other socio-cognitive factors like self-efficacy to delay sex, and knowledge concerning HIV and condom use appeared to be less influential for the motivation to stay abstinent, but were still significant predictors among specific subsamples used in the current study. Addressing motivational factors should preferably be done before sexual debut, since model fit and the amount of significant predictors was lower among adolescents that had already experienced debut. Skill-based and volitional factors such as self-efficacy seem influential after sexual debut, which implies they should be addressed around the time of debut. Finally, future studies that assess socio-cognitive models in relation to sexual behaviour among adolescents in South Africa are recommended to look for disrupting factors, such as economic deprivation or alcohol intoxication, which may explain the relatively large intention-behaviour gap found in this study.
